# Telomerase Dysregulation in the Hippocampus of a Rat Model of Depression: Normalization by Lithium

**DOI:** 10.1093/ijnp/pyv002

**Published:** 2015-03-06

**Authors:** Ya Bin Wei, Lena Backlund, Gregers Wegener, Aleksander A Mathé, Catharina Lavebratt

**Affiliations:** Department of Molecular Medicine and Surgery, Neurogenetics Unit, Karolinska Institutet, Stockholm, Sweden (Mr Wei, Drs Backlund and Lavebratt); Center for Molecular Medicine, Karolinska University Hospital, Stockholm, Sweden (Mr Wei, Dr Lavebratt); Centre for Psychiatric Research and Education, Karolinska Institutet, Clinic for Affective Disorders, Stockholm, Sweden (Drs Backlund and Mathé); Translational Neuropsychiatry Unit, Department of Clinical Medicine, Aarhus University, Aarhus, Denmark (Dr Wegener); Centre of Excellence for Pharmaceutical Sciences, North-West University, Potchefstroom, South Africa (Dr Wegener); and Department of Clinical Neuroscience, Section for Psychiatry,Karolinska Institutet, Stockholm, Sweden (Dr Mathé).

**Keywords:** depression, animal model, lithium, telomerase, telomere, hippocampus

## Abstract

**Background:**

Telomeres are protective DNA-protein complexes at the ends of each chromosome, maintained primarily by the enzyme telomerase. Shortening of the blood leukocyte telomeres is associated with aging, several chronic diseases, and stress, eg, major depression. Hippocampus is pivotal in the regulation of cognition and mood and the main brain region of telomerase activity. Whether there is telomere dysfunction in the hippocampus of depressed subjects is unknown. Lithium, used in the treatment and relapse prevention of mood disorders, was found to protect against leukocyte telomere shortening in humans, but the mechanism has not been elucidated. To answer the questions whether telomeres are shortened and the telomerase activity changed in the hippocampus and whether lithium could reverse the process, we used a genetic model of depression, the Flinders Sensitive Line rat, and treated the animals with lithium.

**Methods:**

Telomere length, telomerase reverse transcriptase (*Tert*) expression, telomerase activity, and putative mediators of telomerase activity were investigated in the hippocampus of these animals.

**Results:**

The naïve Flinders Sensitive Line had shorter telomere length, downregulated *Tert* expression, reduced brain-derived neurotrophic factor levels, and reduced telomerase activity compared with the Flinders Resistant Line controls. Lithium treatment normalized the *Tert* expression and telomerase activity in the Flinders Sensitive Line and upregulated β-catenin.

**Conclusion:**

This is the first report showing telomere dysregulation in hippocampus of a well-defined depression model and restorative effects of lithium treatment. If replicated in other models of mood disorder, the findings will contribute to understanding both the telomere function and the mechanism of lithium action in hippocampus of depressed patients.

## Introduction

Mammalian telomeres consist of tandem repeat DNA sequences (TTAGGG) and protective proteins at each chromosome end, preventing the chromosome from degrading or fusing with other chromosomes (Chan and Blackburn, 2004; Palm and de Lange, 2008). Telomere length (TL) varies between cell types (Friedrich et al., 2000), but the majority of studies have explored TL in peripheral blood leukocytes. Telomeres shorten with each cell division and are suggested to be an indicator of biological aging (Benetos et al., 2001; O’Donovan et al., 2011). Significantly, accelerated shortening occurs in chronic disease states (eg, cardiovascular disease, diabetes, and cancer) with inflammation and metabolic stress, which through oxidative stress damage the telomeres (Price et al., 2013; Verhoeven et al., 2013). In recent years, a number of studies associated shorter blood leukocyte telomere length (LTL) with psychological stress, major depression, and posttraumatic stress disorder (Lung et al., 2007; Wolkowitz et al., 2011; Wikgren et al., 2012; Garcia-Rizo et al., 2013; Verhoeven et al., 2013). With regard to psychological stress and LTL, the results are not uniformly consistent; for example, no association was found between objectively recorded early-life stress (separation from parents) or self-reported significant stress across the life-span and LTL, and only in subjects reporting the combination of both factors could an association be ascertained (Savolainen et al., 2014). Further, Shalev and coworkers (2014) recently showed that persistent early internalizing disorder predisposed LTL shortening in men, but not in women, independently of childhood maltreatment. Accordingly, a large meta-analysis found that females have consistently longer telomeres compared with males (Gardner et al., 2014).

On the other hand, patients diagnosed with schizophrenia were found to have longer leukocyte telomeres compared with controls (Nieratschker et al., 2013). The authors suggested that a possible explanation could be use of psychotropic drugs, not controlled for in their cohort, that have antioxidative and thereby protective effects on the telomeres (Lung et al., 2007; Wolkowitz et al., 2011; Wikgren et al., 2012; Garcia-Rizo et al., 2013; Verhoeven et al., 2013). Interestingly, lithium, the drug of choice in the treatment and relapse prevention of mood disorders, was found to protect against LTL shortening in humans, but the mechanisms have not been elucidated (Martinsson et al., 2013).

Shorter telomeres may result from excessive attrition due to decreased telomerase activity. Telomerase is a ribonucleoprotein consisting of a catalytic subunit with reverse transcriptase activity (TERT) and an RNA subunit (TERC) that serves as a template for DNA synthesis. TERT expression is stringently regulated and of the several splicing forms the full-length mRNA correlates positively with telomerase activity (Kaneko et al., 2006; Bollmann, 2013). Telomerase counteracts the telomere shortening by adding TTAGGG repeats to the chromosome ends (Blackburn and Collins, 2011). In addition to maintaining TL, telomerase is involved in other biological activities, most prominent being mitochondria protection from oxidative stress, DNA repair, antiapoptosis, stimulation of cell proliferation, and stem cell activation (Bollmann, 2008; Cong and Shay, 2008). In the adult rodent and human brains, telomerase is expressed mainly in regions where adult neurogenesis occurs, such as the subgranular zone of the hippocampus (Hermann et al., 2006). TERT also plays important roles in neuroprotection (Fu et al., 2000; Wolf et al., 2011; Li et al., 2013), and it was recently shown that disruption of the telomerase activity in mouse hippocampus led to depression-like behavior, which could be rescued by the antidepressant fluoxetine and by *Tert*-expressing viral vector injection, coupled with the upregulation of telomerase activity (Zhou et al., 2011). In a small open-label study of 16 depressed outpatients treated with sertraline for 8 weeks, there was no overall effect of treatment on telomerase activity. However, those with both low pretreatment telomerase activity and large increase in leukocyte telomerase activity exhibited the largest response to treatment (Wolkowitz et al., 2012). Lithium was previously shown to inhibit glycogen synthase kinase-3β (GSK-3β) (Pasquali et al., 2010), which results in retention of β-catenin (Gould et al., 2004). Lithium-induced upregulation of β-catenin was shown to upregulate *hTERT* transcription in cancer cell lines (Zhang et al., 2012). Lithium has also been reported to promote expression of brain-derived neurotrophic factor (BDNF) which, in turn, enhanced *Tert* expression (Fu et al., 2002).

While shorter telomeres in leukocytes were reported to be associated with major depression, it is not clear whether the same holds true for their respective brains. Two studies (Teyssier et al., 2010; Zhang et al., 2010) reported normal TL in occipital cortex and cerebellum, respectively, of postmortem brains from major depression patients. Szebeni et al. (2014) showed that oligodendrocytes but not astrocytes from depressed individuals displayed shorter TL and reduced *hTERT* expression compared with corresponding postmortem white matter from control brains. *Tert* transcript is highly conserved between human and rodents (Kaneko et al., 2006), thus enabling translational studies in rodent models. The Flinders Sensitive Line (FSL) is a genetic rat model of depression-like behavior and is often compared to the Flinders Resistant Line (FRL). The FSL rats display characteristics that resemble human depression with good face validity, including psychomotor retardation, circadian rhythm disturbances, and cognitive impairment (Overstreet et al., 2005; Overstreet and Wegener, 2013), and have been extensively used to study antidepressant effects of both pharmacological and nonpharmacological treatment modalities, such as antidepressants, ECS, physical activity, and deep brain stimulation (Bjornebekk et al., 2005, 2010; Jimenez-Vasquez et al., 2007; Eriksson et al., 2012; Melas et al., 2012; Rea et al., 2014).

In light of the above, we asked the questions whether telomeres are shortened and the telomerase activity changed in the depressed hippocampus and if so, whether lithium would reverse the process. We attempted to answer these questions by using the FSL rats and treated the animals with lithium. First we investigated if the telomeres were shorter in the hippocampus of the FSL rats, compared with FRL, and if that co-occurred with disturbance of *Tert* expression and telomerase activity. Second, since hippocampi from the FSL rats showed reduced levels, we investigated if lithium treatment would affect these telomere-related measures in the FSL rats. Finally, we investigated expression levels of putative mediators, β-catenin, and BDNF, of lithium’s effect on telomerase activity, both in naïve FSL/FRL and vehicle-/lithium-treated FSL.

## Methods

### Animals and Lithium Treatment

Male FSL and FRL rats were kept under controlled conditions of temperature (22±1°C), relative humidity (45–55%) and daylight cycle (12:12h, lights on at 6:00 am). Normal rat chow and tap water were available ad libitum. A group of FSL rats was randomly assigned to a 6-week treatment with either 2.19g Li_2_SO_4_/kg or vehicle admixed to the rat chow. The lithium-treated rats showed no overt symptoms of toxicity; normal grooming and sleeping behavior were observed. The experimental design was based on our previous studies; under such conditions, lithium serum concentration is within the therapeutic range (Husum et al., 2001; Angelucci et al., 2003). Hippocampi from all the rats were dissected and immediately stored at -80°C until subsequent analyses. All experiments met the guidelines by the Danish National Committee for Ethics in Animal Experimentation and the Ethical Committee for Protection of Animals at the Karolinska Institutet.

### DNA/RNA Extraction and Reverse Transcription

Genomic DNA and total RNA were extracted by AllPrep DNA/RNA/miRNA Universal Kit (Qiagen; Qiagen, Hilden, Germany) and concentrations were determined using the NanoDrop ND-1000 (NanoDrop Technologies Inc.,). Complementary DNA was synthesized using the SuperScript III First-Strand Synthesis System for RT-PCR (Invitrogen; Life Technologies, Carlsbad, CA) according to the manufacturer’s protocol. In brief, equal amounts of RNA were random-hexamer primed at 25°C for 10 minutes, followed by an incubation with SuperScript III RT at 50°C for 50 minutes and termination of the reaction at 85°C for 5 minutes. DNA/complementary DNA was stored at -20^o^C and RNA at -80^o^C until further processing.

### Gene Expression Analyses

Amplification of target and reference genes was assessed using quantitative real-time polymerase chain reaction (qRT-PCR). All qRT-PCR amplifications were performed in triplicate using Power SYBR Green (Applied Biosystems; Life Technologies) on an ABI PRISM 7900 HT Sequence Detection System (Applied Biosystems) with the following conditions: 95°C for 10 minutes, followed by 40 repeats of 95°C for 15 seconds and 60°C for 1 minute, and a final dissociation stage to monitor amplification specificity. Target genes included telomerase reverse transcriptase (*Tert*), brain-derived neurotrophic factor (*Bdnf*), and catenin, beta 1 (*Ctnnb1*). Two reference genes (glyceraldehyde-3-phosphate dehydrogenase; *Gapdh*, and cyclophilin A; *Ppia*) were used for normalization of data. Relative quantification of gene expression was calculated using the qBase software (version 1.3.4; Hellemans et al., 2007). The tested genes and corresponding primer sequences were (written 5’3’): *Tert* Fw: GCAGCAGCCCAGAGAAGGA; *Tert* Rv: CCTCAGCAGCTGTACCACAT; *Bdnf* Fw: GGCCCAACGAAGAAAACCAT; *Bdnf* Rv: AGCATCACC CGGGAAGTGT; *Ctnnb1* Fw: GAAAATGCTTGGGTCGCCAG; *Ctnnb1* Rv: CGCACTGCCATTTTAGCTCC; *Gapdh* Fw: TCGGTGTGAACGG ATTTGGCCG; *Gapdh* Rv: CCGTTGAACTTGCCGTGGGT; *Ppia* Fw: GGC TGATGGCGAGCCCTTGG; *Ppia* Rv: CGTGTGAAGTCACCACCCTGGC.

### Protein Expression

Protein levels were measured using a modified Western-blot protocol as previously described (Wei et al., 2014). Briefly, following sample homogenization and centrifugation, protein concentrations were measured using the Pierce BCA Protein Assay Kit (Thermo Scientific; Thermo Fisher Scientific Inc., Rockford, IL). After incubation at 95°C for 5 minutes, equal amounts of protein (30 µg) were loaded on a NuPAGE Novex 4 to 12% Bis-Tris Gel (Invitrogen). The separated protein was transferred to Amersham Hybond ECL Nitrocellulose Membrane (GE Healthcare; GE Healthcare UK Limited) at room temperature for 1.5 hour and then blocked with 5% nonfat milk for 1 hour at room temperature. Immunoblotting was performed overnight at 4°C with a monoclonal rabbit anti-beta catenin (β-catenin) antibody (1:20000 dilution; ab32572, Abcam; Abcam plc, Cambridge, UK), a monoclonal rabbit anti-BDNF antibody (1:1000 dilution; ab108319, Abcam) and, separately, with a mouse monoclonal anti-β-actin antibody (1:10000; A5316, Sigma-Aldrich, Sigma-Aldrich Co., St. Louis, MO). After washing, the membrane for detecting β-catenin and BDNF was incubated with HRP-linked goat anti-rabbit secondary antibody (1:100000 for β-catenin, 1:50000 for BDNF; Santa Cruz Biotechnology; Santa Cruz Biotechnology Inc., Santa Cruz, CA) and the membrane for detecting β-actin was incubated with HRP-linked goat anti-mouse secondary antibody (1:100000; Santa Cruz Biotechnology) for 1 hour at room temperature. Finally, immunoreactive bands were visualized with the Amersham ECL Plus Western Blotting Detection System (GE Healthcare), exposed to Amersham Hyperfilm ECL (GE Healthcare), and optical densities were quantified using the NIH ImageJ software (1.47 version). β-Catenin and BDNF protein levels were normalized to the levels of β-actin, and the data were presented as relative quantifications.

### Telomerase Activity

The telomerase activity was detected by real-time telomeric repeat amplification protocol (RT-TRAP) (Hou et al., 2001) with some modifications. In brief, the rat hippocampus was lysed in CHAPS buffer, and the total protein concentration was measured by the Pierce BCA Protein Assay Kit (Thermo Scientific; Thermo Fisher Scientific Inc., Rockford, IL). Equal amount of protein (1.0 µg) from each sample was added to a reaction mix with a total volume of 25 µL containing 2.5mM of each dNTP, 20mM Tris-HCl (pH 8.3), 2.5mM MgCl_2_, 63mM KCl, 0.05% Tween 20, 1mM EGTA, 0.1mg/mL BSA, and 0.1 µg each of the primers TS (5’-AATCCGTCGAGCAGAGTT-3’) and ACX (5’-GCGCGG(CTTACC)3CTAACC-3’). A HeLa cell line was used as a telomerase-positive control, whereas CHAPS buffer and heat-inactivated samples were used as negative controls. TSR8 is an oligonucleotide with a sequence identical to the TS primer extended with 8 telomeric repeats being AG(GGTTAG)_7_. Serial dilutions of TSR8 control template were used to generate a standard curve to calculate telomerase activity. The serial dilutions were 0.2 amoles/μL, 0.04 amoles/μL, 0.008 amoles/μL, 0.0016 amoles/μL, and 0.00032 amoles/μL, corresponding to 200, 40, 8, 1.6 TPG units/μL; TPG is the Total Product Generated, corresponding to the number of TS primers (1 unit = 10^–3^ amoles or 600 molecules) that are extended with at least 3 TTAGGG repeats by telomerase in the extract in a 30-minute incubation at 30°C. The reaction mix was incubated at 30°C for 30 minutes followed by termination at 95°C for 5 minutes. Then 10 µL of the telomeric repeat products was used for the real-time telomeric repeat amplification protocol assay amplified by Power SYBR Green. The reaction was performed on ABI PRISM 7900 HT Sequence Detection System with the following conditions: 95°C for 10 minutes, followed by 36 repeats of 95°C for 20 seconds, 52°C for 30 seconds, and 72°C for 60 seconds.

### Telomere Length Measured by qRT-PCR

Relative TL of the rat hippocampal DNA was determined according to the protocol of Cawthon et al. (2002). In brief, triplicate DNA samples (4.0ng) were used both for the telomeres (Tel) and the single-copy gene (ribosomal protein L30, *Rpl30*) qRT-PCR, which was performed within the same 384-well plate, amplified by using Power SYBR Green in 10 µL total volume. The reaction was performed on ABI PRISM 7900 HT Sequence Detection System with the following conditions: 95°C for 10 minutes, followed by 39 repeats of 95°C for 15 seconds and 60°C for 1 minute, followed by a dissociation stage to monitor amplification specificity. The relative TL was calculated according to the 2^-ΔΔCt^ method, where ΔΔCt =ΔCt_sample_-ΔCt_calibrator sample_ and ΔCt_sample_=Ct_Tel_-Ct_single copy gene_. The tested genes and corresponding primer sequence were (written 5’3’): Tel1: CG GTTTGTTTGGGTTTGGGTTTGGGTTTGGGTTTGGGTT; Tel2: GG CTTGCCTTACCCTTACCCTTACCCTTACCCTTACCCT; *Rpl30* Fw: CA GACGCCAAGATGGCCGGG; *Rpl30* Rv: GCTCGGCTTCTGCTTCCGCT

### Statistical Analyses

Data in the bar graphs are presented as mean values ± 1 SEM. Normality of the data and the homogeneity of the variance were tested using Shapiro-Wilk and Levene’s tests, respectively. The difference in mean between 2 groups was assessed using 2-tailed Student’s *t* test. The threshold for statistical significance was set at *P*<.05. All analyses were performed using IBM SPSS Statistics version 22 (IBM Corporation, Armonk, NY).

## Results

### Shorter Telomeres, Decreased *Tert* Expression and Telomerase Activity, and Decreased BDNF Expression in the Hippocampus of the Naïve FSL Rats

**Figure 1.**
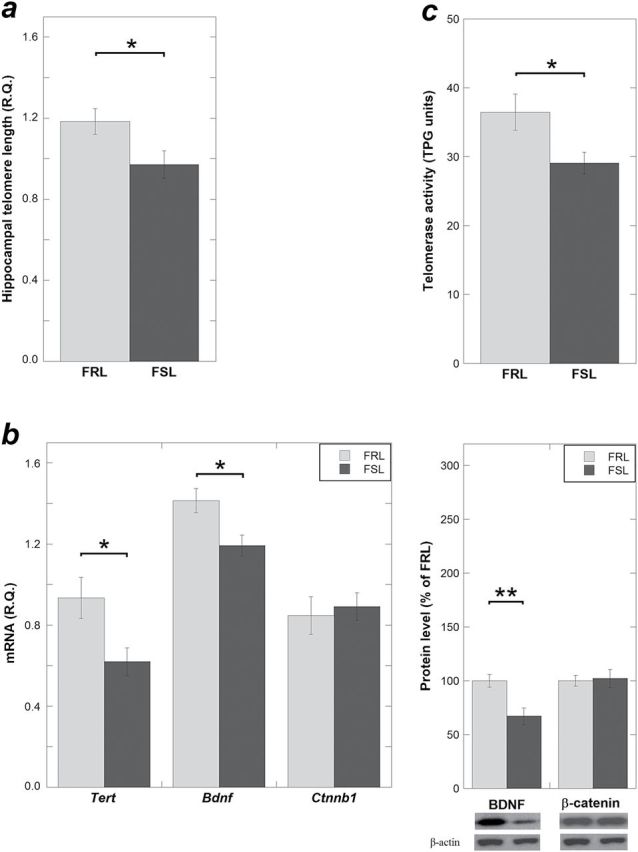
Telomere length (TL), *Tert*, brain-derived neurotrophic factor (BDNF), β-catenin expression, and telomerase activity in hippocampus of naïve Flinders Sensitive Line (FSL)/Flinders Resistant Line (FRL) rats. (*a*) Naïve FSL rats had shorter telomere length compared with the same age FRL rats, measured using quantitative real-time polymerase chain reaction (qRT-PCR). (*b*) *Tert* and *Bdnf* mRNA levels were reduced in the FSL compared with the FRL rats, measured using qRT-PCR (left panel). BDNF protein levels were reduced in FSL rats (right panel). (*c*) Telomerase activity was reduced in naïve FSL, detected by real-time telomeric repeat amplification protocol. Telomere length and gene expression data are presented as relative quantifications (R.Q.). Protein data are presented as percent of FRL. Lower right panels in (*b*) show representative Western-blot images of BDNF and β-catenin with β-actin as loading control. Telomerase activity is presented as TPG units. (a: n = 11 FRL, n = 16 FSL; n = 2 FSL outliers excluded; b-c: n = 6–8 animals per group; n = 1 FSL outlier excluded). Data are presented as means ± SEM, *P< .05, **P < .01.

First, we measured the TL in hippocampi from the FSL and FRL rats. The FSL had shorter TL compared with the FRL (*P*=.038) (Figure 1a). Second, we explored if the shorter telomeres in the FSL could reflect a reduced telomerase activity. Since the expression levels of full-length *Tert*, in most cases, correlate with telomerase activity (Greenberg et al., 1998; Kaneko et al., 2006), we determined the expression levels of the full-length *Tert. Tert* levels were reduced in the FSL compared with the FRL rats (*P* = .023) (Figure 1b). Consistent with the downregulation of *Tert* expression, telomerase activity was lower in the depressed FSL (*P* = .041) (Figure 1c). Finally, BDNF both expression and protein levels were significantly lower in the FSL hippocampi compared with FRL (mRNA: *P* = .023 and protein: *P* = .007) (Figure 1b), which was in agreement with the decreased telomerase activity in FSL.

### Lithium Treatment Increases *Tert* and Telomerase Activity and β-Catenin Expression in the Hippocampus of the FSL

**Figure 2.**
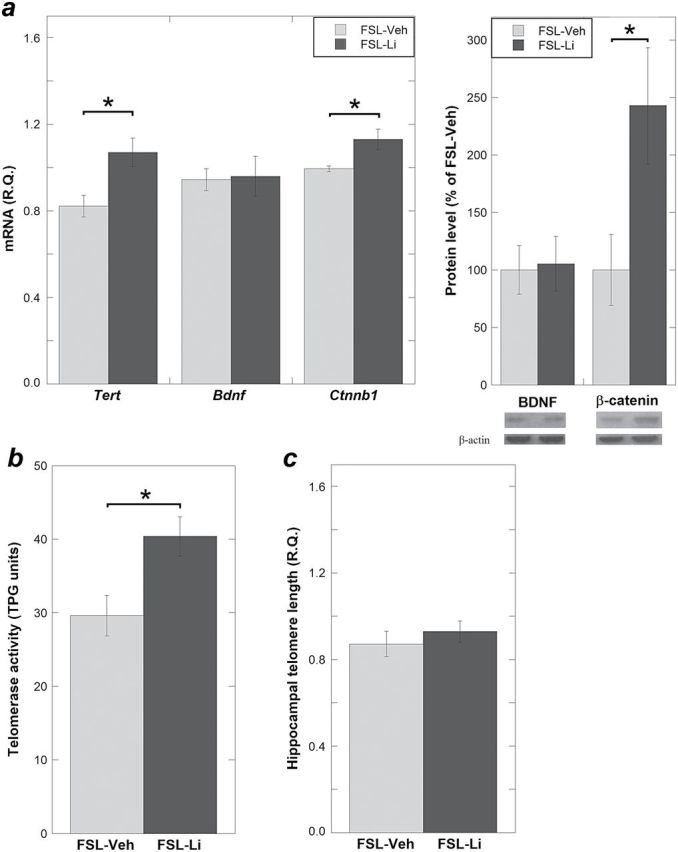
Expression levels of *Tert*, brain-derived neurotrophic factor (BDNF), β-catenin, telomerase activity, and telomere length (TL) measured in the hippocampus of vehicle-treated Flinders Sensitive Line (FSL) (FSL-Veh) and lithium-treated FSL (FSL-Li). (*a*) *Tert* mRNAs that showed different expression levels in hippocampi comparing naïve FSL with FRL were significantly upregulated in the FSL-Li group compared with FSL-Veh. This was associated with β-catenin upregulation both at mRNA (left) and protein (right) levels. (*b*) Consistent with the upregulation of *Tert* levels in the FSL-Li group, telomerase activity was also increased. (*c*) The point estimate of the telomere length mean was increased in the FSL-Li group; however, it was not significantly longer than in the FSL-Veh group. Gene expression data are presented as relative quantifications (R.Q.). Protein data are presented as percent of FSL-Veh. Lower right panels in (*a*) show representative Western-blot images of BDNF and β-catenin with β-actin as loading control. Telomerase activity is presented as TPG units. n = 6–8 animals per group; n=1 outlier was excluded in Figure 2a from each group; data are presented as means ± SEM, * *P* < .05.

*Tert* expression was increased in the hippocampi from the lithium-treated FSL (FSL-Li) compared with the FSL vehicle-treated group (*P* = .012) (Figure 2a). In line with the *Tert* upregulation, telomerase activity was also increased in the FSL-Li group (*P* = .015) (Figure 2b). To test the hypothesis that β-catenin mediates lithium’s effect on *Tert*, we measured the β-catenin levels in FSL-Veh and FSL-Li hippocampi. No baseline differences between naïve FSL and naïve FRL were found (mRNA: *P* = .31 and protein: *P* = .83) (Figure 1b). However, lithium significantly increased β-catenin expression in FSL both at mRNA and protein levels (mRNA: *P* = .028 and protein: *P* = .036) (Figure 2a). Additionally, we explored if lithium influenced the hippocampal TL in the FSL rats; the point estimate of the TL mean was increased in the FSL-Li group but did not reach the statistical level of significance (*P* = .46) (Figure 2c). Lastly, BDNF levels were not influenced by lithium (mRNA: *P* = .89 and protein: *P* = .87) (Figure 2a).

## Discussion

We provide 2 novel findings on telomere regulation in a well-documented model of depression. First, hippocampal telomeres were shorter in the FSL rats compared with the control rats. In accordance with this finding, the *Tert* expression and telomerase activity were reduced in the FSL hippocampus, which was in agreement with a decreased BDNF expression in FSL. Second, the aberrant *Tert* expression and telomerase activity in the FSL hippocampus were reversed after lithium treatment at clinically relevant doses. A possible mediator of this effect was β-catenin, which was upregulated by lithium treatment.

### Disturbed Hippocampal TL and Telomerase Activity in a Rat Model of Depression

Shorter blood LTL has been associated with depression and posttraumatic stress disorder in several studies (Lung et al., 2007; Wolkowitz et al., 2011; Wikgren et al., 2012; Garcia-Rizo et al., 2013; Verhoeven et al., 2013). However, it is not known whether telomeres are also shorter in the brain of depressed subjects. In an attempt to answer this question, we used a reversed translational strategy and examined brains of FSL rats, a genetic rodent model of depression. Since the hippocampus plays pivotal roles in cognitive function (Sweatt, 2004), mood regulation, and memory formation (Becker and Wojtowicz, 2007), we assessed TL in that region and found that FSL had shorter TL than control rats. As telomerase is an important determinant of TL, we studied that enzyme and found that its activity was decreased in hippocampi from FSL rats.

Telomerase overexpression has been suggested to promote adult neurogenesis in the hippocampus (Wolf et al., 2011; Zhou et al., 2011). Substantial literature shows the existence of adult neurogenesis, particularly in the dentate gyrus (Christian et al., 2014), and the role of reduced neurogenesis in the pathophysiology of depression (Campbell and Macqueen, 2004; Lee et al., 2013). Interestingly, chronic mild stress in mice resulted in decreased TERT levels and telomerase activity and reduced neurogenesis in hippocampus, as well as depression-like behavior. In contrast, fluoxetine and intrahippocampal injection of an adenovirus vector expressing *Tert* reversed these effects, leading the authors to suggest that hippocampal telomerase plays a role in depression-like behaviors, possibly by regulating neurogenesis (Zhou et al., 2011). We investigated the full-length *Tert* transcript that encodes functional telomerase (Kaneko et al., 2006) in the hippocampus of depressed FSL rats and found that it was decreased. Correspondingly, telomerase activity was also reduced in FSL, which was in line with the work by Zhou et al (2011). BDNF was reported to modulate telomerase activity in embryonic hippocampal neurons (Fu et al., 2002); thus, the decreased BDNF levels we observed in the naïve FSL may in part underlie its reduced telomerase activity. On the other hand, the *Tert* expression difference between naïve FSL and FRL may also be related to genetic variation between these strains in telomere regulating genes, or genes implicated in metabolic stress, inflammation, or oxidative stress. For example, we previously found that the FSL harbors a functional 1-base genetic variant in the promoter of the neuropeptide Y (*Npy*) gene, which modulates *Npy*’s transcriptional activity (Melas et al., 2013). In humans, a number of single nucleotide polymorphisms in telomere-regulating genes have been associated with LTL (Codd et al., 2013; Oddsson et al., 2014). Cortisol levels were suggested to influence telomerase activity (Choi et al., 2008; Gotlib et al., 2014); however, serum corticosterone levels were not different between FSL and FRL rats (Owens et al., 1991; Ayensu et al., 1995). The telomerase deficiency in the FSL hippocampus implicated potential disturbance in cell survival and proliferation; this warrants future investigation.

### Lithium Treatment Increases Hippocampal Telomerase Activity

In addition to the documented efficacy in treatment and prophylaxis of mood disorders (Johnson et al., 2001; Husum and Mathé, 2002; Angelucci et al., 2003; Miller et al., 2007), therapeutic effects of lithium have been explored in several degenerative CNS disorders, notably Alzheimer’s disease, amyotrophic lateral sclerosis, and stroke (Chiu and Chuang, 2011). On the cellular level, lithium exerts a variety of facilitatory and inhibitory effects on enzymes and signaling systems; for instance, it enhances neuroprotective pathways, for example, Bcl-2 and Wnt signaling, and inhibits phosphatidylinositol phosphatases and GSK-3, (α and β). Inhibition of GSK-3β is proposed to be a core mediating event of neuroprotective and neurotrophic effects of lithium (Pasquali et al., 2010). Importantly, β-catenin, an established marker for GSK-3β inhibition (Gould et al., 2004), was shown to be involved in activation of *hTERT* transcription in cancer cell lines (Zhang et al., 2012). However, molecular mediators of lithium’s effect on telomerase have not been clarified. Our results show that lithium rescued the reduced *Tert* expression and telomerase activity in the FSL hippocampus. In line with these results, lithium significantly increased β-catenin expression. This is the first report on the possible mechanism of lithium’s modification of hippocampal TL. Lithium was previously reported to upregulate BDNF levels in hippocampus and temporal cortex (Fukumoto et al., 2001; Hashimoto et al., 2002). However, we found no BDNF increase in hippocampi from lithium-treated FSL measured by Western blot, which is consistent with our previous results measured by ELISA (Angelucci et al., 2003). This discrepancy in effect on BDNF might be explained by differences in the duration of lithium treatment. In the study of Fukumoto et al. (2001), an increase in BDNF was found after 14 days, but not 28 days, while the treatment duration in our study was 42 days. In this study, lithium did not increase hippocampal TL, that is, the TL did not follow the change in telomerase activity. A similar lack of TL change despite telomerase upregulation was reported by Wolkowitz et al. (2012). TL changes much slower than telomerase activity (Epel et al., 2009; Epel et al., 2010), and our previous study found that long-term lithium treatment (≥30 months) in patients diagnosed with bipolar disorder correlated positively with LTL (Martinsson et al., 2013). Species and tissue differences, that is human vs rodent and brain vs leukocytes, are possible explanations for these discrepancies.

Limitations of our study are that we did not perform behavioral tests comparing the FSL-vehicle and FSL-Li treated groups and therefore cannot provide direct evidence that enhanced telomerase activity by lithium is associated with an antidepressant-like effect. Moreover, due to the insufficient number of available FRL rats, we did not compare possible differential effects of lithium on FSL and FRL rats. On the other hand, the primary aim of the lithium treatment was to verify our hypothesis that lithium protects against TL shortening and to explore the possible molecular mechanisms. Another limitation is that our results were derived from hippocampus homogenates; thus, cell type-specific expression should be addressed in follow-up studies. For example, telomerase activity was recently reported to be expressed not only in neural stem cells but also in astrocytes and oligodendrocytes in white matter of the adult brain (Szebeni et al., 2014). The strength of this project is that we demonstrated, for the first time, that 1) telomeres are shorter, 2) *Tert* expression is reduced, 3) telomerase activity is decreased in hippocampus of a rat genetic model of depression, and 4) lithium treatment increases expression of β-catenin and *Tert* and telomerase activity in the FSL rat hippocampus.

## Interest Statement

None.
